# Circular RNA-Mediated Regulation of Oral Tissue-Derived Stem Cell Differentiation: Implications for Oral Medicine and Orthodontic Applications

**DOI:** 10.1007/s12015-024-10683-w

**Published:** 2024-01-27

**Authors:** Tudor-Sergiu Suciu, Dana Feștilă, Ioana Berindan-Neagoe, Andreea Nutu, Gabriel Armencea, Alexandra Iulia Aghiorghiesei, Talida Vulcan, Mihaela Băciuț

**Affiliations:** 1 Department of Orthodontics and Dentofacial Orthopedics, Iuliu Hațieganu University of Medicine and Pharmacy, 400083 Cluj-Napoca, Romania; 2 Research Center for Functional Genomics, Biomedicine and Translational Medicine, Iuliu Hațieganu University of Medicine and Pharmacy, 400337 Cluj-Napoca, Romania; 3 Department of Maxillofacial Surgery and Implantology, Iuliu Hațieganu University of Medicine and Pharmacy, 400029 Cluj-Napoca, Romania; 4 Department of Prosthodontics and Dental Materials, Iuliu Hațieganu University of Medicine and Pharmacy, 400006 Cluj-Napoca, Romania; 5 Department of Dermatology, Iuliu Hațieganu University of Medicine and Pharmacy, 400006 Cluj-Napoca, Romania

**Keywords:** Circular RNAs, Regulatory function, Tissue engineering, Osteogenic Differentiation, Periodontal ligament stem cells, Oral medicine

## Abstract

Circular RNAs (circRNAs) are a novel class of endogenous non-coding RNAs (ncRNAs) which unlike linear RNAs, have a covalently closed continuous loop structure. circRNAs are found abundantly in human cells and their biology is complex. They feature unique expression to different types of cells, tissues, and developmental stages. To the present, the functional roles of circular RNAs are not fully understood. They reportedly act as microRNA (miRNA) sponges, therefore having key regulatory functions in diverse physiological and pathological processes. As for dentistry field, lines of evidence indicate that circRNAs play vital roles in the odontogenic and osteogenic differentiation of dental pulp stem cells (DPSCs) and periodontal ligament stem cells (PDLSCs). Abnormal expression of circRNAs have been found in other areas of pathology frequently reflected also in the oral environment, such as inflammation or bone and soft tissue loss. Therefore, circRNAs could be of significant importance in various fields in dentistry, especially in bone and soft tissue engineering and regeneration. Understanding the molecular mechanisms occurring during the regulation of oral biological and tissue remodeling processes could augment the discovery of novel diagnostic biomarkers and therapeutic strategies that will improve orthodontic and other oral therapeutic protocols.

**Graphical Abstract:**

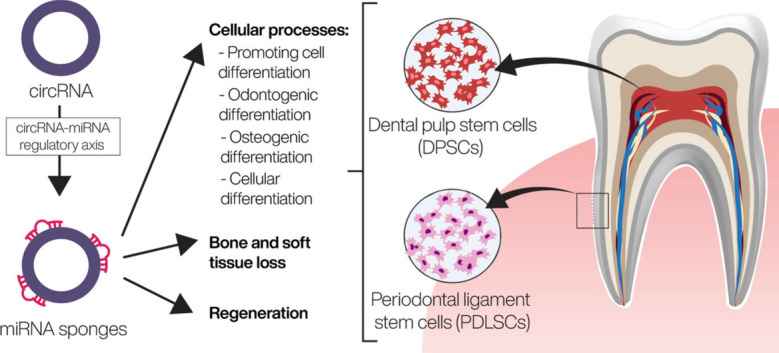

## Introduction

Modern orthodontic therapy can be enhanced with the help of circular RNAs (circRNAs), as they take part in the mechanical induction of alveolar bone remodeling. CircRNAs also have significant roles in other processes in the oral environment. Oral pathologies like inflammation, bone or soft tissue loss, and cancers are currently major health issues worldwide. These diseases can also cause systemic affliction. The molecular mechanisms underlying these oral pathologies must be explored to determine relevant diagnostic biomarkers and therapeutic targets [1].

Circular RNAs represent a novel class of endogenous non-coding RNAs (ncRNAs) which possess a covalently closed continuous loop structure [2]. Due to the advances in high-throughput sequencing technology [2], circRNAs have been identified to be widely expressed in eukaryotic cells**.** Although at first they were considered non-functional accidental by-products of aberrant splicing [3], circular RNAs (circRNAs) were identified in mammalian cells in the 1990s [4]. The importance of these circRNAs did not receive attention in the biological field until several recent studies reported the large presence of circRNA in many different cell lines of humans. It was not until then that their biological functions were discovered [5]. Due to the head-to-tail closed loop structure from 3’ end to 5’ tail [6], circRNAs are not susceptible to digestion by ribonucleases (RNases), thus possessing higher stability than linear RNA and remaining constant between species [7]. Moreover, studies have shown that the sequences of circRNAs exhibit a wide distribution in the organism and have high evolutionary conservation while also displaying cell/ tissue/developmental‑stage‑specific expression.

An expanding number of studies suggest that circRNAs play critical roles in cellular functions. All these findings led to the rational supposition that the existence of altered expression patterns of circRNAs in different tissues and fluid body compartments might represent an act of cause or effect for a multitude of human diseases [8], including inflammatory response, with circRNAs combining with other noncoding RNAs such as microRNAs (miRNAs) [9, 10].

CircRNAs were found to exist constantly in tissues, in the cellular nucleus and cytosol, and on extracellular exosomes. In body fluids [11], they are considered ideal diagnostic biomarkers and therapeutic targets with vital research significance and clinical value [12].

## Biogenesis

The biogenesis of circRNAs is, up to now, uncertain [2]. Many studies have shown that the synthesis of circRNAs is characterized by a unique, non-canonical type of splicing known as back-splicing [13, 14]. A 3’ splice acceptor is joined to an upstream 5’ splice donor to generate a circular structure [15]. This looping is mediated by the pairing of bases between inverted repeat elements, by dimerization of RNA-binding proteins or by binding of RNA-binding proteins to specific motifs in the flanking introns, as these bring a downstream splice-donor site close to an upstream splice-acceptor site [16]. The biogenesis of circRNAs is characterized by a lower back-splicing efficiency as compared to that of canonical splicing. One possible explanation is that the exons and spliceosomes are detrimentally massed at back-splicing sites to catalyze the bound of upstream 3’ acceptor sites with downstream 5’ donor sites, even in the presence of the canonical splice sites [2].

**Fig. 1:**
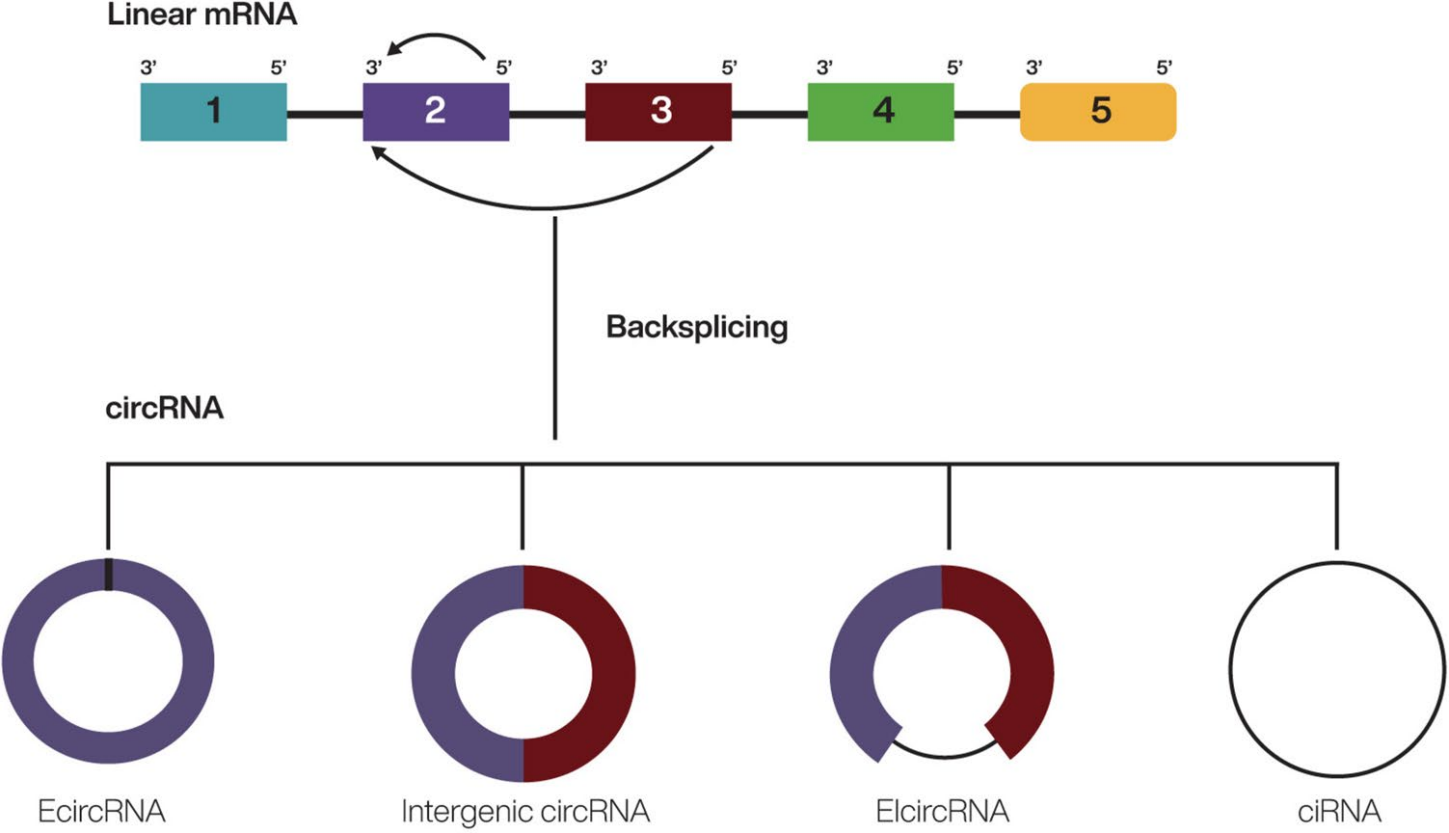
The biogenesis of circular RNAs, created via back-splicing CircRNAs are constructed by a particular, non-canonical type, known as back-splicing. The diverse mechanisms for connecting exons and introns in back-splicing generate different circRNAs. Colored bars – exons; black line – introns. Figure created with BioRender.com

Several mechanisms for connecting exons and introns in back-splicing have been described. The different mechanisms for connecting exons and introns in back-splicing lead to different types of circRNAs (Fig. 1) [17].

One crucial hypothesis is that formation of circRNAs is due to base pairing between reverse complementary sequences, often repetitive ALU elements, and flanking introns [4]. Intronic repeat arrangements bind to one another, bringing the intervening splice sites close to each other, thus promoting circular RNA production [17]. According to a second model for the biogenesis of circRNAs, circularization is believed to be mediated by RNA binding proteins (RBP). RBPs promote the genesis of circRNAs by regulating adjacent splice sites [14, 18]. Splicing factors such as Quaking and Muscleblind activate circularization by binding to sequence motifs of flanking introns, linking two flanking introns close together and bringing the circularized exons nearby [18]. The mechanism is comparable to intron-pairing-driven circularization, the difference being that RNA binding proteins control neighbouring splice sites rather than a direct base pairing between complementary motifs [19]. Additionally, the immune factors NF90 and NF110 contain double-stranded RNA-binding domains, fuse with intronic RNA pairs, stabilize these pairs and augment the expression of circ-RNAs [18, 20]. On the opposite, adenosine deaminase and ATP-dependent RNA helicase A destabilizes RNA pairing and therefore inhibit circRNAs expression [16, 21]. Similarly, the RNA-editing enzyme ADAR1 inhibits circRNAs formation by binding to double-stranded RNA and melting the stem structure [19].

A third mechanism, exon skipping or lariat-driven circularization, involves a linear exon-containing lariat precursor formed during canonical RNA splicing. When the lariat undergoes internal back-splicing, the flanking intronic sequences are removed and a circular structure is produced [16]. When, under some circumstances, these sequences are retained, an exon–intron circRNA results. Circular intronic RNA formation is also facilitated by this mechanism [18].

The biogenesis of circRNAs can be directly influenced by epigenetic changes within histones and gene bodies. For instance, methylation of the gene body can upregulate or downregulate circRNA expression. At their turn, circRNAs can influence the state of the promoter of their host genes epigenetically [16].

Circular RNAs biogenesis is tightly regulated. The regulatory mechanisms are not yet fully understood. The regulatory rules of circRNA biogenesis are dependent on the cis-regulatory elements and trans-acting factors which control splicing involved in the splicing process. One important control factor seems to be intronic complementary repeats in intron flanking the circularized exons [2]. Other factors are proteins involved in the production of circRNAs, such as RNA-binding proteins, RNA helicase, spliceosome components and even RNA Pol II [2, 22].

After formation, most circRNAs are exported to the cytoplasm, where they are predominantly located. Only intron-containing circRNAs remain within the nucleus [16]. Even if the back-splicing has low efficiency, as mentioned above, circRNAs accumulate to high levels due to their resistance to degradation [14]. Circular RNAs can also be detected in body fluids, such as blood, urine and saliva [23].

## CircRNAs in Dental Pulp Tissue

The dental pulp consists of an extracellular matrix and of cells, among which are fibroblasts, odontoblasts, immune cells and endothelial cells. The functions of dental pulp are complex and include the formation and nourishment of dentin protection and teeth restoration. Odontoblasts carry out dentin formation, but the interaction between different types of cells within the pulp and between cells, vascularization, and innervation is also important for dental pulp formation and homeostasis. Teeth that have lost pulp or in which the pulp has been destructed no longer can form dentin and therefore lose vitality and become vulnerable.

To maintain vitality of teeth, tissue engineering-based approaches for dental pulp regeneration are considered promising therapies [24, 25]. Human dental pulp stem cells (hDPSCs) are multipotent mesenchymal stem cells originating in dental pulp tissues. They are characterised by self-renewal capacity, a high proliferative potential and multi-lineage differentiation, which allow hDPSCs to differentiate into neurogenic, osteogenic and dentinal cell lineages, depending on different conditions [6, 26]. Due to their characteristics and the fact that, unlike other sources of mesenchymal stem cells, dental pulp stem cells collection implies minimal trauma, hDPSCs represent a potential candidate for the regenerative treatment of dentin loss and bone defects [27, 28]. However, biological processes such as osteogenic differentiation, odontogenesis and pulp regeneration are not fully understood and therefore need further studying.

Studying the molecular mechanisms underlying these processes is of significant importance to understand the biology of pulp regeneration, dentin and bone formation. To this respect, investigating the regulatory roles of circRNAs and their effects on human dental pulp stem cells has become a research subject of great importance, which could reveal new biomarkers and potential targets for the regenerative treatment using mesenchymal stem cells.

Several publications demonstrated that during odontogenesis and osteogenesis of teeth-derived stem cells, circRNAs are differentially expressed and participate in tissue regeneration and stem cell differentiation [7]. By containing miRNA binding sites, circRNAs usually function as miRNA sponges. CircRNAs involved in regulating these biological processes are hsa_circRNA_104101, circRNA12453, hsa_circ_0015260 and hsa_circ_0006984, circAKT3, circLPAR1, circSIPA1L1 and has_circ_0026827. They support odontogenesis, as well as osteoblast differentiation, by sponging miRNAs.

In an attempt to shed light on the potential role of circRNAs in hDPSCs during odontogenic differentiation, as well as to provide a new theoretical foundation for the study of pulp regeneration, Chen et al. [12] studied the differential expression of circRNAs in hDPSCs during odontogenic differentiation. They detected the expression profiles of 12,929 circRNAs. Of the 187 circRNAs that presented a significantly different word in the differentiated state compared to the undifferentiated one, 43 were upregulated and 144 downregulated. To further continue the study, one predicted circRNA was selected based on the results to study its effect on odontogenesis. The authors found that downregulation of hsa_circRNA_104101 led to decreased expression of genes involved in odontoblastic differentiation, such as dentin sialophosphoprotein (DSPP) and dentin matrix protein-1 (DMP1), or in those that indicate osteoblastic differentiation such as DMP1, alkaline phosphatase (ALP) and osteocalcin (OCN). Therefore, they suggested that hsa_circRNA_104101 and its target genes were correlated with the odontogenic differentiation of hDPSCs.

Based on the circRNA-miRNA-mRNA network construction using bioinformatics analyses, it was predicted that DSPP and DMP1 might be correlated to has_miR-708-5p and has-miR-642a-5p, which in turn may be related to the differentially expressed circRNAs during hDPSC odontogenic differentiation.

The study aimed to shed light on the potential role of circRNAs in odontogenesis, as well as to provide a new theoretical foundation for the study of pulp regeneration. It was also discovered that hsa_circRNA_104101 was involved in the odontogenic differentiation of human dental pulp stem cells, hinting at the possible role of circRNAs as new therapeutic targets.

Another circRNA that seems to be associated with the osteogenic differentiation of DPSCs both in vitro and in vivo is circRNA124534 [29]. The biological process involved appears to be the Wnt/β-catenin signaling pathway, which is known to play a vital role in the osteogenic differentiation of DPSCs [30]. CircRNA124534 sponges miR-496 during DPSCs osteogenic differentiation and so reverses its repressive function on the β-catenin target. Because the interaction of miR-496 with the 3’ UTR of β-catenin suppresses its activity at the post-transcriptional level, circRNA124534 proves a vital role in the development of osteogenesis and could be seen as a potential therapeutic target for bone regeneration.

Other circRNAs that reinforce odontogenesis by sponging miRNAs are hsa_circ_0015260 and hsa_circ_0006984. By sponging miR-135b, their increase in the study of Chen Li and Hongwei Jiang [7] releases the inhibition of the miRNA mentioned above on its target genes competitively, ultimately enriching MAPK signaling pathway.

One of the miRNAs that inhibit osteogenesis in osteoblasts and MSCs is miR-206, which targets the 3’UTR region of connexin 43 (CX43) mRNA and downregulates its expression [31]. CX43 is expressed in multiple cell types and plays a critical role in osteogenesis and odontogenesis. [32, 33]. As for hDPSCs, circAKT3 was the most upregulated circRNA during hDPSCs osteogenic differentiation, and it removed inhibition of CX43 and subsequently suppressed odontogenesis [34].

Along the same lines regarding the osteogenic differentiation of DPSCs, Xingyun Ge and his collective suggested that the circSIPA1L1/miR-617/Smad3 axis is a critical factor in stimulating DPSC osteogenesis [6]. Thus, one downstream gene for miR-617 is circSIPA1L1, which promotes osteogenesis by positively regulating ALP, the transcription regulator for early-stage osteogenesis RUNX2 and key downstream genes of RUNX2, namely Osterix (OSX), a zinc finger-containing transcription factor that is essential in osteogenesis differentiation and hence for bone formation [35] and Smad3, a vital component of the TGF-β signaling pathway that participates in bone formation [36]. By downregulating and neutralising miR-617, the upregulated circSIPA1L1 favors hDPSCs osteogenesis, with the circSIPA1L1/miR-617/Smad3 axis playing a vital role in the process.

In their recent study, Ji et al. [37] demonstrated the involvement of hsa_circ_0026827 in the osteogenic differentiation of DPSCs. Hsa_circ_0026827 acts as a sponge for miR-188-3p and removes its inhibitory effect on RUNX1, a key transcription factor that takes part in different maturational processes required for skeletal development, [38] and on Beclin1 3’UTRs, which mediates autophagy. It was previously discovered that autophagy has essential roles in the stemness of MSC and can stimulate their osteogenic differentiation [39]. Taken together, the findings suggest that hsa_circ_0026827 promotes osteogenic differentiation as its expression was increased during the process, and by downregulation of the circRNA, osteoblast differentiation was suppressed. A new insight on the mechanisms underlying osteogenic differentiation is provided by demonstrating that hsa_circ_0026827 promotes osteoblast differentiation of DPSCs through the Beclin1 and RUNX1 signaling pathways by sponging miR-188-3p. Furthermore, the data offered by the study indicate new therapeutic strategies for the regeneration of bone.

For hDPSCs to be successfully used in regenerative therapies, they must preserve their properties. One important factor affecting cell proliferation by causing genomic instability, cellular senescence and stem cell apoptosis is oxidative stress [40, 41]**.** Reactive oxygen species (ROS) are generated through various mechanisms within the human body. They have a role in regulating physiological and biological functions, such as intracellular signaling, regulation of homeostasis or induction of death [40]. Oxidative stress resulting from exacerbated ROS production exerts deleterious effects on biological macromolecules. Past research has established that a number of the dysregulated functions of stem cells under oxidative stress were reversible by overexpression of specific genes or by targeting critical signaling pathways. [40]. Roles of ROS in stem cells and circRNAs related to oxidative stress have only lightly been touched. It has been reported that ROS regulate the balance between both embryonic and adult stem cell self-renewal and differentiation, which is critical for tissue homeostasis. To maintain the function of stem cells, regulation of the oxidation–reduction state (redox) must be considered [42].

The latest studies are reporting that a decisive role in generating oxidative stress and in the post-transcriptional gene regulation in oxidative stressed stromal cells might be played by circRNAs [43]. To this respect, Zhang et al. [41] have found 330 upregulated, and 533 downregulated circRNAs in hDPSCs exposed to oxidative stress.

Furthermore, hsa_circ_0000257 was upregulated while hsa_circ_0087354 and has_circ_0001946 were downregulated, all associated with the post-transcriptional regulation of oxidative stress in hDPSCs. These circRNAs sponge miRNAs as follows: the upregulated hsa_circ_0000257 regulated 128 microRNAs, while hsa_circ_0087354 and hsa_circ_0001946 regulated 58 and 123 microRNAs. Bioinformatics and KEGG analyses revealed that p53 and MAPK signaling pathways, cell cycle and serotonergic synapse were involved in modulating oxidative stress. It has been previously suggested that P53 signaling pathway is a possible key mediator of oxidative stress in hDPSCs [44]. It seems that P53 is the downstream gene of sirtuin1 (SIRT1), and its deacetylation in adipose-derived stromal cells attenuates apoptosis [45]. In the study of Zhang and his collaborators, hsa_circ_0000257 sponges several miRNAs, like hsa-miR-9–5, which share highly conserved complementary sequences with SIRT1. Other studies in different cell systems have confirmed that SIRT1 is a direct target gene of hsa-miR-9–5. Wang’s group demonstrated that miR-9-5p inhibition attenuated MPP + -induced cell apoptosis, inflammation and oxidative stress by directly targeting SIRT1 [46]. A connection between miR-9, SIRT1 and oxidative stress regulation has also been identified by D’Adamo et al. in human primary and C-28/12 chondrocytes [47]. All these lines of evidence prove that specific circRNAs are involved in the oxidative stress of hDPSCs, like hsa_circ_0087354 and hsa_circ_0001946 being downregulated and hsa_circ_0000257 being upregulated in the oxidatively stressed cells. P53 signaling pathway could play a role in oxidative stress, with hsa_circ_0000257/ hsa-miR-9-5p/ SIRT1/ P53 regulatory axis representing a possible molecular pathway regulating oxidative stress in hDPSCs. Targeting such critical signaling pathways to reverse dysregulated functions of stem cells under oxidative stress can be of therapeutic interest [41].

An emerging field of study is represented by the roles of exosomal RNAs in various biological processes. Different RNAs contained in exosomes represent vital contributors to their functions. Exosomal microRNAs showed altered expression during osteogenic differentiation [48]. Circular RNAs, which have displayed changes in expression during osteogenic differentiation, are also contained in exosomes. Nevertheless, the subject of exosomal circRNAs has only lightly been studied so far, and their role in osteogenesis requires further investigations.

Xie et al. [49] examined exosomal circRNAs after osteogenic induction of DPSCs. They found circLPAR1 to be constantly upregulated throughout the process. The roles of circLPAR1, a G-protein coupled receptor found in normal human tissues, on osteogenesis have yet to be elucidated. Bioinformatics analyses reported a robust binding capacity of circLPAR1 for hsa-miR-31. It was previously proven that hsa-miR-31 inhibited the osteogenic differentiation of mesenchymal stem cells by targeting SATB2, a protein with a critical role in bone biology and positively correlated with osteogenic markers like RUNX2 [50]. With their study, Xie and his collective confirmed that the expression levels of SATB2 and other osteogenic differentiation-related genes were upregulated in the circLPAR1-overexpressed groups.

Thus, it was brought to attention that circLPAR1 was strongly expressed in the exosomes of osteogenic-induced DPSCs. Binding to hsa-miR-31, the micro RNA that targets SATB2, circLPAR1 eliminated its negative effect on the osteogenic differentiation of DPSCs. Finally, SATB2 expression increased, leading to the upregulation of its downstream genes, such as RUNX2. In turn, RUNX2 promoted osteogenic differentiation occurrence and development. The findings of this study shed light on the expression profile of exosomal circRNAs during osteogenic differentiation of DPSCs. They unveiled one of the roles played by exosomes during the process. This brings us one step closer to understanding the mechanisms of osteogenic differentiation of DPSCs.

**Fig. 2:**
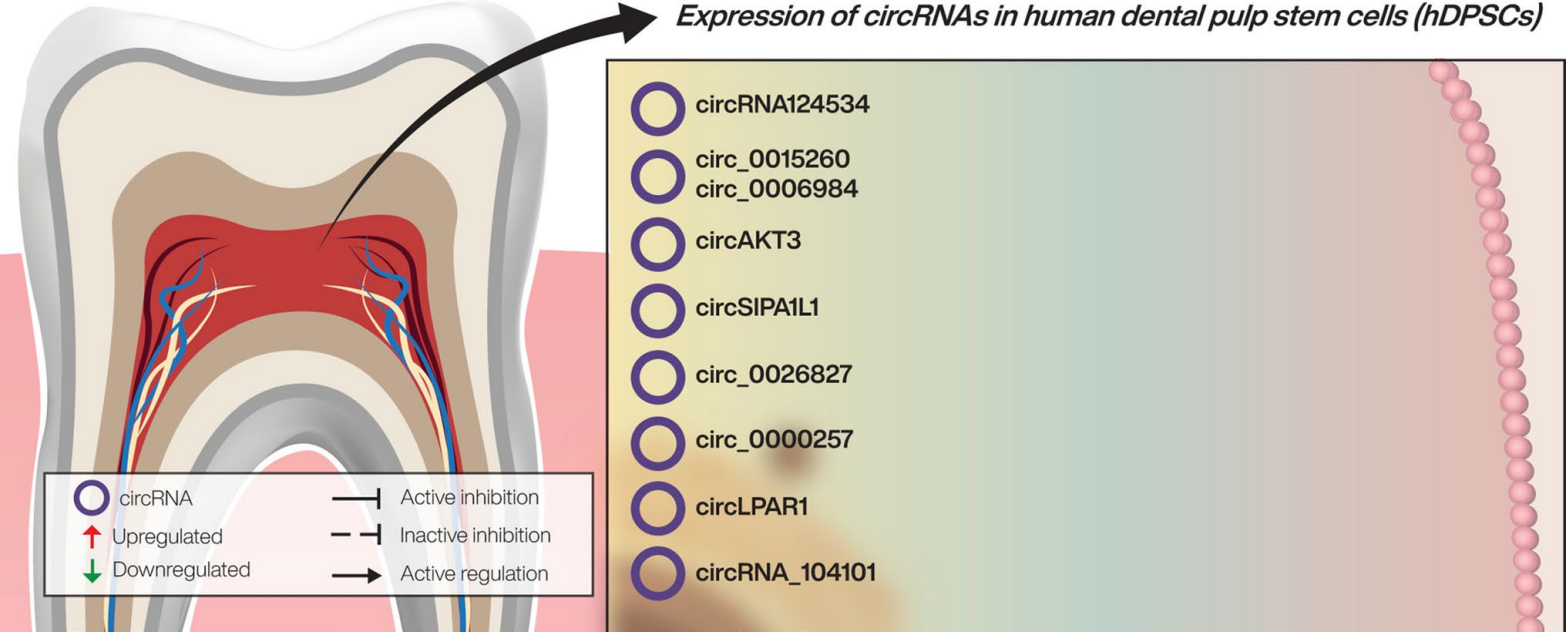
Illustrated overview of circRNAs expression in hDPSCs. According to different studies, expression of circRNA and regulation of downstream components usually follow circRNA-miRNA-mRNA regulatory network, being one of the main molecular mechanisms. As result, critical signaling pathways are targeted, such as MAPK, β-catenin, p53, or Wnt pathways. Nevertheless, there are still some unknown mechanisms, which should be elucidated, as in the case of the regulatory mechanism of circRNA_104101

In this respect, contemporary studies have demonstrated the presence of circRNAs in hDPSCs isolated from dental pulp tissues and their involvement in odontogenesis and osteoblast differentiation of hDPSCs by sponging miRNAs (Fig. 2). Further research is required to fully acknowledge their functions and effects on dental pulp stem cell differentiation and uncover the molecular mechanisms by which such products are exerted. Such studies could provide potential targets for regenerative treatment and bone remodeling therapies using mesenchymal stem cells.

## CircRNAs in Periodontal Ligament Stem Cells and Periodontal Tissue Regeneration

In conditions that produce destruction of the periodontium, regeneration cannot be achieved predictably using current therapies. Under such circumstances, tissue engineering has a good potential for periodontal regeneration [51]. The periodontal ligament is a soft tissue that supports the teeth, cushions masticatory force, and aids in tooth nutrition, alveolar bone restructuring and orthodontic tooth movement [9]. Human periodontal ligament stem cells (PDLSCs) are derived from periodontal ligament tissue and are considered the best source for periodontal tissue regeneration [51]. PDLSCs are appropriate for osteogenic tissue engineering [52, 53]. Their potential resides in their properties, such as multipotency, high osteogenic potential and immunomodulation, and also in the fact that they can be easily obtained [51]. Understanding the molecular pathways behind PDLSCs osteogenic differentiation could contribute to discovering periodontal regenerative therapies.

The capability of PDLSCs to differentiate into osteoblasts was reportedly associated with circRNAs in previous in-depth studies [5, 54]. Mechanisms involved in bone regeneration may be related to circRNAs-miRNAs-mRNAs relations. MicroRNAs function by post-transcriptionally repressing protein manufacturing or triggering mRNA degradation. CircRNAs have been proven to regulate miRNAs by acting as sponges or decoys, thus shortening the number of available miRNAs [14].

A vital contributing factor in periodontal tissue regeneration is represented by the stemness of PDLSCs, including their self-renewal capacity, proliferation, and differentiation potential to osteoblasts, fibroblasts and cementoblasts. In an attempt to explore the regulatory genes of stemness, Gu et al. demonstrated that CDR1as, an antisense transcript of cerebellar degeneration-associated protein 1 (CDR1), enhances the osteogenic differentiation of PDLCs and maintains their cell migration ability [55]. Regarding CDR1 targets, it is known that it has approximately 70 conserved miR-7 binding sites and acts as a miR-7 sponge [56]. It has been indicated that osteoblastic differentiation of PDLSCs was regulated via the miR-7/HDF5/SMAD and p38 MAPK signaling pathways [9]. The expression levels of stemness-related markers, such as SOX2, OCT4 and Nanog, were increased by CDR1 overexpression, while the knockdown of CDR1 decreased them [55]. These findings of Gu et al. are consistent with previous observations, as they reported that CDR1 promoted the pluripotent state of PDLSCs by diminishing miR-7 mediated suppression of Kruppel-like factor 4 (KLF4) expression. KLF regulates cell fate by directly modulating the transcription of underlying genes [57]. In PDLSCs, KLF4 knockdown decreases the expression levels of stemness-related markers SOX2, OCT4 and Nanog in PDLSCs. The study conducted by Gu et al. hints at a new genetic strategy for PDLSC-based periodontal regenerative medicine [55].

Recently, RNA populations, including mRNAs and non-coding RNAs such as circRNAs and miRNAs, were discovered in exosomes [13, 58]. Exosomes are one of the significantly characterized subpopulations of extracellular vesicles. They can interact with cells by acting as messenger vehicles, transferring information which may modulate the phenotype of target cells [59, 60]. In the recent past, exosomes have been associated with bone regeneration [61] and therefore exploring exosomes would be of interest in regard to the osteogenic differentiation of cells. Xie et al. [5] demonstrated essential changes in exosomal circRNAs at the early phase of osteogenic differentiation of PDSCLs. Their study found that circRNAs were expressed in exosomes to communicate with and influence nearby or remote cells. In this respect, previous studies have also indicated that MSCs might act as paracrine to control tissue regeneration [62]. Of hundreds of circRNAs differently expressed, few were validated through RT-qPCR. Only hsa_circ_0087960 (circRNA lysophosphatidic acid receptor 1, LPAR1) was firmly confirmed, being increasingly expressed after 5 and 7 days of osteogenic induction [5].

### Orthodontic Movement

Orthodontic tooth movement (OTM) involves mechanical induction of bone remodeling. During the process, alveolar bone reconstruction is determined by different biochemical signals induced by mechanical force (MF) [63, 64]. Tensile strains cause bone formation, while compressive strains mediate bone resorption. PDLSCs control these MF and the periodontal ligament recovery process when such forces are removed, playing a vital role in the alveolar bone remodeling during MF-dependent OTM [65].

Mechanical force changes crucial signal pathways at the transcription level in PDLSCs and promotes the osteogenic differentiation of PDLSCs [63, 64]. In addition, the properties of PDLSCs, *e.g.*, immunomodulation, homeostasis, and remodeling of the periodontal ligament, allow these cells to play an essential role in tooth movement. It has been suggested that MSCs might act as paracrine to control tissue regeneration [62]. Indeed, the biological insights have also highlighted PDLSCs as promising immune-modulator agents.

While knowledge on the molecular mechanisms of orthodontic tooth movement has constantly improved, data in recent years have suggested a role for non-coding RNAs in modulating processes involved in orthodontic tooth movement. Chen and Zhang [66] mentioned changes in ncRNA expression profiles in cell cultures in animal models and indicated that differential miRNA expression profiles could be attributed to types, intensities and durations of orthodontic forces. Jiao et al. [17] found non-coding RNA expression to be different in compressed PDLSCs as compared to normal cells. These data reinforce other studies that suggest miRNAs could be mechanosensitive [67]. Li et al. [67] also cited several papers indicating that miR-21, miR-34a stimulated osteogenic differentiation of PDLSCs exposed to mechanical forces. They added that different types of forces might induce different miRNA expression profiles.

If this is the case, differences in miR expression during orthodontic tooth movement could reflect their different effects on the molecular processes involved. More than this, Chen and Zhang [66] also indicated that a single miRNA could interact with many other mRNAs and that this works both ways. These observations, taken together with the fact that miRNAs are regulated by circRNAs could lead to the idea that circRNAs have to play an important part in OTM. However, data published so far on this aspect are scarce.

As previously mentioned, circRNAs play a key role in stem cell differentiation. Gu et al. [68] identified 1382 circRNAs that could bind with 148 common miRNAs and also predicted these miRNAs to regulate the pluripotency of PDLSCs. It has also been shown that, during PDLSC osteogenic differentiation, circRNA expression is stage specific [69].

Regarding to circRNAs expression in tissues exposed to mechanical force data in scientific literature have shown a sum of circRNAs being over- or underexpressed in skin exposed to tension [70], in osteoblasts exposed to microgravity [71] or related to mechanical forces in cartilage and joint surfaces of patients with osteoarthritis [72]. Data regarding hard tissues within the oral cavity were brought by Wang et al. [73], who described a novel molecular mechanism in bone environment as a response to tension force. The authors identified in their recent study a new circular RNA, namely circ_0008542, located in tension force-induced osteoblast exosomes. This circRNA, by competitively binding with miR-185-5p, upregulated its target gene RANK and initiated bone resorption through osteoclast differentiation. What’s more, circ_0008542 increased its sponge effect on miR-185-5p by increasing its expression profile with prolonged tension stimulation time, confirming the observations of Chen and Zhang [66].

The response of circRNAs in orthodontic tooth movement is rather unknown. Scientific literature in recent years indicated that mechanical forces associated with OTM induce changes in circRNA expression in PDLSCs with possible consequences upon PDLSCs osteogenesis induced by such forces [66, 74]. According to these authors, circRNAs might play essential roles in morphological changes of the cells and osteogenic differentiation of PDLSCs under mechanical stimulation. In the study of Wang et al. [74], 2678 significantly diffferentially expressed circRNAs were identified in mechanical force-induced PDLSCs. While thousands of circRNAs seem to be dysregulated in such circumstances, bioinformatics analysis highlighted circRNA4214, circRNA3140, circRNA5331, circRNA126, circRNA4045, circRNA4251 overexpression as being associated with osteogenesis of PDLSCs exposed to MF. All these circRNAs act by binding miRs and affecting their posttranscriptional modulation. CircRNA4214, which was highlighted as significantly upregulated in MF-induced PDLSCs, regulates the mitogen-activated protein kinase MAPK signaling pathway. As Wang et al. pointed out, circRNA4124 took part in different molecular processes such as mechanical stimulus–response, cell adhesion and migration, inflammatory response, immune response, protein binding. Others have also indicated MAPK to be one of the mechanical signaling pathways that have a crucial role in osteoblast proliferation in response to mechanical strain [75].

CircRNA3140 was found to be highly associated with miR-21, known to exert roles in MF-induced osteogenic differentiation of PDLSCs [76] and to regulate Toll-like receptor (TLR) signaling pathway, which can affect the osteogenic potential of human mesenchymal stromal cells [77]. CircRNA5331, by sponging miR-204, can cancel its negative regulation of Runt-related transcription factor 2 and remove its inhibition of osteogenesis of mesenchymal stem cells. Furthermore, circRNA126, circRNA4045 and circRNA4251 were predicted to interact with miR-101, miR-335 and miR-107, respectively [74]. The authors also mentioned that exposure to mechanical force led to an increase in the expression profile of circRNA3154, circRNA5034, circRNA3133 and circRNA5045 and that these changes are associated with extracellular vesicular exosomes and Wnt signaling pathway [74]. Earlier reports showed that by targeting the Wnt/β-catenin signaling pathway, miR-101 and miR-335 regulate the osteogenic differentiation of mesenchymal stem cells [74, 78].

Other circRNAs are downregulated in PDLSCs exposed to mechanical forces. More than 1400 circRNAs with a downregulated expression profile were found by RNA sequencing and 2 of these, circRNA1818 and circRNA1358, were validated by RT-qPCR analysis [74]. The targets and interactions of these circRNAs are unclear so far. Last but not least, circRNA436 presented a significantly downregulated expression in the stretched PDLSCs and was found to be related to both miR-107 and miR-335, indicating that it could be a key regulator of MF-stimulated PDLSC differentiation. It has been shown that miR-107 is involved in the differentiation of several cell types [79, 80]. As for miR-335, it is believed that its targets include genes that regulate cellular movement and gene expression, such as RUNX2, involved in osteogenic differentiation [81]. These observations indicate that circRNA436 could be a key regulator of MF-stimulated PDLSC differentiation.

CircRNAs exhibit substantial regulatory roles in the process of orthodontic tooth movement, showing potential as both biomarkers and therapeutic agents. Their involvement in orthodontics is constantly being discovered, promoting the transition of research findings from the laboratory to clinical practice. Potential uses of circRNAs encompass investigating the most effective orthodontic force, averting unwanted side effects and enhancing clinical results in order to achieve desired treatments. Exploring delivery systems for ncRNA-based therapies, along with identifying and validating circRNAs and their molecular pathways and target genes might lead to obtaining personalized orthodontic treatments.

### Periodontal Disease

Inflammation, loss of soft tissues/teeth/bone, and other oral disorders have become major public health concerns worldwide. These problems not only impair local function but can also lead to the development of various systemic diseases. As a result, it is critical to investigate the underlying molecular pathways causing these oral disorders to develop useful diagnostic biomarkers and treatment targets [1].

The healthy periodontium consists of soft tissues, namely the gingiva and periodontal ligament, hard tissues, cementum and alveolar bone. When a microbial biofilm forms at the junction of the tooth and the neighboring gingiva, it causes localized inflammation of the gingiva, named gingivitis [17]. If not treated promptly, gingivitis can evolve into periodontitis, which implies that inflammation can extend deep into tissues. Besides this, periodontitis leads to abnormal vascularization. The result is ischemia and hypoxia of periodontal tissues and subsequently irreversible periodontal tissue degradation and alveolar bone loss [82]. As a result, the repair of the bone defect is thought to be essential to periodontal regeneration [83], and in this process, PDLSCs are essential. However, an important factor to be considered is the presence of inflammation and hypoxia in periodontal disease. The properties, along with the possible underlying intracellular mechanisms of PDLSCs depend on their interaction with the surrounding inflammatory microenvironment when inflammation is the reason for tissue damage [84].

Many studies suggest that circRNAs may play critical roles in regulating immune-inflammatory responses. Although little is known about the underlying mechanisms of circular RNAs in periodontal tissues and their regulatory functions under inflammatory and hypoxic circumstances, there is an emerging understanding of their potential role. The primarily accepted mechanism is, as in any other case, that circRNAs regulate miRNAs expression profile via acting as miRNAs sponges and further regulating the expression of functional genes [9]. CircRNAs found in inflamed tissue from periodontitis and being associated with PDLSCs properties are circMAP3K11 [83], has_circ_0003489 [85], circ_0095812 and circ_0062491 [10], CDR1as [86].

In their study, Jie li and Ruiyue Xi [10] investigated circular RNAs in gingival tissues and the circRNAs-miRNAs interaction mechanism in periodontal disease and they discovered 1304 abnormally expressed circRNAs. Data indicated that circ_0095812 and circ_0062491 might have vital roles in the pathobiology of periodontitis. These 2 circRNAs interacted with many miRNAs, and miR-584 and miR-128 were involved in the process. About miR-584, it is known that its P. gingivalis-dependent upregulation reduced the anti-inflammatory response by decreasing the level of lactoferrin receptor in gingival epithelial OBA-9 cells [87]. Downregulation of circ_0062491 leads to overexpression of miR-584 in THP-1 cells exposed to P.Gingivalis. Others found MiR-128 to be a critical factor in regulating the excessive inflammatory response to minimize tissue damage by mediating endotoxin tolerance through p38 MAPK pathway modulation [88].

Wang et al. investigated the role of circRNA CDR1 in the proliferation of PDLSCs and the mechanisms involved. To this purpose, an inflammatory condition was induced by P gingivalis-derived LPS, which is a pertinent deleterious factor in the oral microenvironment. The study indicated that circRNA CDR1as mediates the process of periodontitis and potentially influences the functions or characteristics of PDLSCs. CDR1as was downregulated in PDLSCs under inflammatory conditions. CircRNA knockdown enhanced the LPS-induced proliferative inhibition of PDLSCs, whereas CDR1as overexpression mitigated this inhibition effect. As mentioned in another section of this article, the circRNA regulates the proliferation of PDLSCs by sponging miR-7. While osteoblastic differentiation of PDLSCs is controlled via the miR-7/HDF5/SMAD and p38 MAPK signaling pathways [9], in inflammation induced by P. gingivalis, CDR1as activated the ERK signaling pathway to promote PDLSC proliferation, emphasizing the functional roles of CDR1as/miR-7 in PDLSCs in an inflammatory state [86].

In a recent study, Yu et al. [83] found that circMAP3k11 (hsa_circ_002284) was involved in periodontitis development in mice by sponging miR-511-3p and regulating Toll-like receptor 4 (TLR4) expression. It has been previously demonstrated that miR-511 can regulate immune functions and that one of the studied target genes of miR-511 is TLR4 [89]. TLR4 was formerly proved to mediate the activation of proinflammatory cytokines and play a negative role in periodontitis pathogenesis [90]. CircMAP3k11 also interferes with the stemness properties of PDLSCs, affecting cell proliferation and apoptosis and suppressing the regenerative capacity of these cells. Silencing circMAP3K11 could hinder the viability, expansion, and migration of PDLSCs and cause apoptosis. On the other hand, overexpression of the circRNA led to inverse results and more than this, it reduced the expression of OSX, which is one of the proteins associated with osteoblast differentiation, via its upstream transcriptional activators RUNX2 and ATF4.

Another circRNA that affects chronic periodontitis development is hsa_circ_0003948 [91]. The circRNA was downregulated in gingival tissues from periodontitis patients, while its overexpression alleviated LPS-induced PDLC injury by sponging miR-144 and removing its inhibitory effect on NR2F2/ PTEN signaling. Phosphatase and tensin homolog deleted on Chromosome 10 (PTEN) is a negative regulator of a significant cell growth and survival signaling pathway, namely the phosphatidylinositol-3-kinase (PI3K)/AKT signaling pathway [92].

Regarding hypoxic influences on the osteogenic differentiation of PDLSCs, Zheng et al. [85] found a potential network of regulatory relationships between hypoxia, autophagy, endoplasmic reticulum (ER) stress, apoptosis, the mTOR pathways and osteogenesis. Several physiological and pathological processes in human diseases are associated with dysregulated autophagy [93]. The activation of autophagy in inflamed periodontal ligament tissues interrupts the equilibrium between osteogenesis and osteolysis, which results in bone loss. In Zheng’s study, hypoxia upregulated circCDK8 expression, which led to autophagy, osteogenic inhibition and apoptosis promotion in PDLSCs through the mTOR pathway when hypoxia exposure time went beyond 24 h. Hence, circCDK8 is suggested as a potential therapeutic target for the future treatment of periodontitis.

**Fig. 3:**
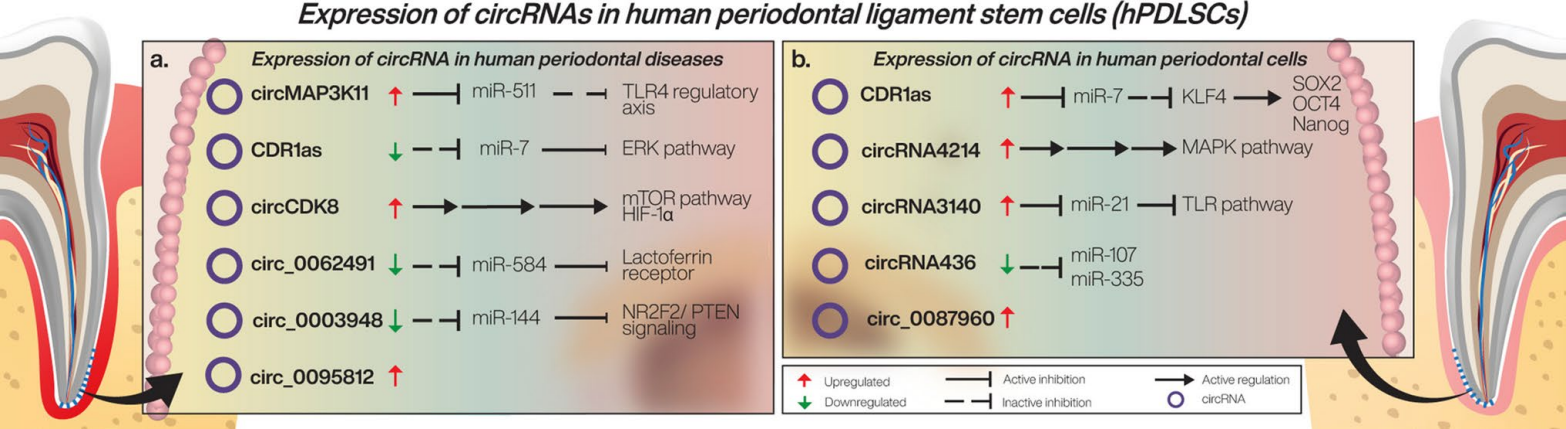
Schematic representation of the circRNA-miRNA networks in hPDLSCs (**A**) Regulatory pathways of circRNAs that are involved in periodontal diseases such as periodontitis (circMAP3K11, CDR1as, circCDK8, hsa_circ_0003948, circ_0095812, and circ_0062491); (**B**) Regulatory pathways of circRNAs that are involved in different mechanisms of PDLSCs such as osteogenesis (circRNA4214, circRNA3140), osteogenic differentiation (circ_0087960, CDR1as), maintenance of cell migration ability (CDR1as), and MF-differentiation of PDLSC (circRNA436)

In summary, recent evidence indicates that the expression of several circRNAs varies throughout PDLSCs differentiation and that certain circRNAs have specific functions in controlling stem cell differentiation (Fig. 3). There is not enough understanding of tissue regeneration and bone remodeling in oral medicine. The detailed mechanisms by which circRNAs regulate stem cell differentiation need more research to understand how they could be used in periodontal regeneration.

## CircRNAs in Maxillary Sinus Membrane Stem Cells and Apical Papilla Stem Cells

Inadequate alveolar bone and the presence of the maxillary sinus at the posterior maxilla represent a contraindication of different dental interventions, like the insertion of dental implants. Maxillary sinus floor elevation is the election procedure in these cases for solving the inadequate bone height issues in the posterior maxilla. On the other hand, maxillary sinus membrane stem cells (MSMSCs) from the maxillary sinus membrane have been reported to be able to differentiate into osteoblasts [94], marking them as a potential source of cells for use in bone tissue engineering.

Various paracrine factors strictly control MSMSC differentiation. Bone morphogenetic protein 2 (BMP2) is a member of the transforming growth factorβ (TGF-β) superfamily, which participates in bone formation and remodeling. BMP signaling also participates in the differentiation of mesenchymal stem cells into osteoblasts. However, until now, little is known about the functional role of circRNAs during BMP2-induced osteogenic differentiation.

MSMSCs are essential for bone augmentation in sinus lifting procedures and are more and more associated with bone tissue engineering studies [95]. Attempting to characterize these sinus-derived osteoprogenitors and their roles further, Peng et al. [96] studied the differential expression of circRNAs in BMP2-treated MSMSCs. Of the 50 most differentially expressed circRNAs, 32 were upregulated, and 18 were downregulated. CircRNA_33287 was identified to have the highest expression level out of the upregulated circRNAs. It was also found that circRNA_33287 significantly elevated the expression levels of osteogenesis-related factors Runx2, OSX and ALP, promoting the osteogenic differentiation of MSMSCs. The data obtained implied that circRNA_33287 was a key factor for the upregulation of osteogenesis-related factors during the early stage of osteogenic differentiation in MSMSCs.

It has been demonstrated that circRNAs participate in osteogenic differentiation. Moreover, circRNAs have been proven to act as competitive endogenous RNAs. By containing multiple miRNA binding sites, they function as miRNA sponges, enabling them to further regulate gene expression. In the present study, with the help of luciferase reporter assay, Peng et al. identified circRNA_33287 to bind to miR-214-3p directly. Furthermore, they demonstrated that overexpression of miR-214-3p decreased the expression of previously mentioned osteogenic markers (Runx2, OSX and ALP) in osteogenic differentiated MSMSCs. At the same time, the downregulation of miR-214-3p promoted the osteogenic differentiation of the sinus-derived stem cells. MiR-214-3p also targeted Runx3 to suppress the osteogenic differentiation of MSMSCs, which could partially be reversed by Runx3 overexpression. The gain- and loss-of-function experiments conducted in the study showed that circRNA_33287 overexpression led to increases in Runx2, OSX and ALP expression, but miR-214-3p overexpression significantly suppressed these increases. However, the knockdown of miR-214-3p reversed its suppression effect. After circRNA_33287 overexpression in mice transplanted with a Bio-Oss scaffold containing MSMSCs, the formation of new bone was enhanced. These results imply that circRNA_33287 has a functional role of a molecular sponge of miR-214-3p, activating the Runx3 target gene during osteogenic differentiation of MSMSCs both in vitro and in vivo.

The above lines of evidence indicate that BMP2 could induce osteogenic differentiation by stimulating the production of circRNA_33287, which then acts as a sponge for miR-214-3p and thus promotes Runx3 expression. Claiming that their study is the first to associate circRNA_33287 with osteoblast differentiation, the authors further suggest that the circular RNA might be a new therapeutic target for regulating osteoblast differentiation in the posterior maxilla.

The apical papilla consists of soft tissue lightly anchored to the apices of immature permanent teeth. SCAPs, or stem cells from the apical papilla, are a novel type of dental mesenchymal stem cells found in the apical papilla of developing permanent teeth [97]. They possess the characteristics of MSCs, such as high proliferative potential, self-renewal ability, and low immunogenicity [98]. Data suggests that SCAPs can differentiate into various cell lineages, including osteogenic, odontogenic, adipogenic and chondrogenic cells, which marks them as candidates for stem cell-based therapeutic approaches [99].

The promising roles of SCAPs in alveolar bone regeneration, dental pulp and dentin formation have been intensively studied [100]. Furthermore, SCAPs have been shown to have the ability to form and regenerate bone tissue in several earlier investigations [101, 102]. Given the attributes of SCAPs, which lead to their potential in regenerative medicine, understanding their functions and characteristics at the genetic and molecular level are vital [103].

MiRNAs have previously been demonstrated to participate in SCAPs' osteogenic differentiation [104]. By acting as miRNA sponges, many circRNAs are involved in various cell biological processes, including cell osteogenic differentiation modulation via the circRNA-miRNA-mRNA axis. These findings, along with specific characteristics of circRNAs like cell type and gene-specific expression profiles, show that circRNAs could be a novel type of regulator for SCAP differentiation into distinct cell lineages [105]. The role of circRNAs in modulating SCAPs osteogenic differentiation, on the other hand, is still unknown.

In a recent study, Zehan Li et al. [103] set to investigate the expression profiles of circRNAs, miRNAs and mRNAs in osteogenesis-driven SCAPs. They discovered 650 circRNAs had a significant expression change during SCAPs osteogenic differentiation, with 333 circRNAs being upregulated while the other 317 showed downregulated expressions. Bioinformatics analyses were then performed to uncover the possible regulatory roles of the differentially expressed circRNAs, revealing that their host genes were associated with the MAPK signaling pathway. Previous studies have already demonstrated the significant roles of the MAPK pathway in the osteogenic differentiation of SCAPs [106]. Moreover, various biological processes and molecular functions were correlated with the host genes of the differentially expressed circRNAs, like developmental processes and cellular regulation. CircRNA-miRNA-mRNA networks were further predicted using specific bioinformatics software, exposing a complicated relationship between circRNAs and miRNAs, in which one circRNA could influence many miRNAs in diverse ways.

Further experiments on one of the circRNAs from the network, circNFATC1, showed that its knockdown hindered the osteogenic differentiation of SCAPs. On the other hand, as one of the underlying targets of circNFATC1, the miR-4483 expression profile decreased during osteogenic differentiation in SCAPs, while circNFATC1 showed a steady increase. Hence, circNFATC1 was suggested as a potential regulator of SCAPs' osteogenic differentiation by modulating miRNAs and their target genes.

Based on the bioinformatics predictions, another regulatory axis constructed by circSIPA1L1, miR-204-5p, and ALPL was highlighted as having potential roles in the osteogenic differentiation of SCAPs [103]. CircSIPA1L1 was previously demonstrated to promote the osteogenic differentiation of dental pulp stem cells [6]. However, the role of circSIPA1L1 in SCAPs osteogenesis has not been uncovered yet. Yuzhi Li et al. [107], in their recent study, detected the expression profile of circSIPA1L1 during SCAPs osteoblast differentiation, which they found was gradually increasing. Additional findings confirmed that overexpression of circSIPA1L1 promotes osteogenic differentiation of SCAPs in vitro via sponging miR-204-5p.

Nonetheless, the data obtained from the experiments also indicated that it is possible that miR-204-5p is not the only target of circSIPA1L1, suggesting a more complicated role of the circRNA in the regulation of osteogenic differentiation. Regarding miR-204-5p, the results of the current study demonstrated that the miRNA targets the 3’UTR of the ALPL (alkaline phosphatase) gene, inhibiting its translation at the post-transcriptional level. ALPL gene encodes ALP (alkaline phosphatase) protein, which is vital for bone and teeth mineralization. These findings, when taken together, reveal that circSIPA1L1 can enhance the osteogenic differentiation of SCAPs by acting as a miR-204-5p sponge to promote the expression of ALP.

At present, understanding the roles of circRNAs and their underlying molecular mechanisms in SCAPs osteogenic differentiation is just the beginning. Recent research suggests that the expression of different circRNAs varies during SCAPs' osteogenic differentiation, with some of them having particular roles in modulating stem cell differentiation. However, further studies are required to elucidate the complicated processes through which circRNAs regulate SCAPs differentiation and their potential functions in regenerative medicine.

## Conclusions and Perspectives

With the evolution of high-throughput sequencing techniques and bioinformatics analyses, circRNAs gained more consideration of late, which led them to become a prominent research subject. Being widely distributed in the transcriptome, circRNAs participate in various physiological and pathological processes in the organism. The scientific community has generally accepted that circRNAs regulate cell proliferation and differentiation, apoptosis and various signaling pathways, as they have been proven to play essential roles in different tissues.

The research included in this review provided insights into the roles of different circRNAs and their modulatory networks in dental pulp stem cells' odontogenic and osteogenic differentiation. The studies show circRNAs which have an abnormal expression in DPSC during osteogenic and odontogenic differentiation while also playing regulatory roles in promoting cell differentiation. CircRNAs mainly exert their effects on regulating stem cell differentiation by sponging miRNAs, with the circRNA-miRNA regulatory axis being the most investigated molecular mechanism. However, the studies of circRNAs’ roles in cellular differentiation and tissue engineering are still in their early stages. As the essence and value of circRNAs speak for themselves, further research on their functions in stem cell differentiation is crucial.

CircRNAs released from periodontal cells play a critical role in periodontal tissue function, contributing to homeostasis and regeneration or tissue loss during periodontitis. Looking at the expression profiles of circRNAs from diseased tissues, various circRNAs, including circMAP3K11, CDR1as, circ_0062491, circ_0095812 and circCDK8, were differentially expressed in PDL or gingival tissues from sites with periodontitis. Even if these circRNAs are potentially associated with periodontitis, further research is required as minimal data is available on the expression of circRNAs in periodontal tissues.

Most studies investigating the involvement of circRNAs in periodontology concentrated on their effect on cell differentiation in wound healing and tissue regeneration. Still, knowledge of the roles of circRNAs in cellular differentiation and tissue engineering is only beginning. Regarding the studies included in this review in periodontal tissue, most of them focused on the part of different circRNAs in the osteogenic differentiation of periodontal ligament stem cells, with the circRNA-miRNA-mRNA regulatory network being one of the main molecular mechanisms. However, this offers an understanding of only one part of their overall function. Future therapeutic applications will benefit from more excellent knowledge of the molecular pathways participating in bone regeneration or the etiology of periodontal bone loss.

Moreover, this might help identify new molecular elements that could be biomarkers for disease detection or therapeutic targets to treat certain bone-related oral diseases. Considering that PDLSCs are potential cell sources for bone tissue engineering, it is vital to understand how the microenvironment can affect them at a molecular level before implementing intervention strategies. Future research needs to be conducted on other aspects of circRNAs to comprehend the full spectrum of potential mechanisms that underlie their capabilities in tissue regeneration.

Based on the findings included in this review, circRNAs have a role in regulating oral biological processes. They could be a regulatory component in the development and outcome of oral medicine. Research on the roles of circRNAs in oral medicine is also challenging. For example, even if specific circRNA/miRNA/mRNA interactions have been uncovered, numerous circRNAs have more than 1 miRNA-binding site and can target multiple genes. The functional circRNA-parental mRNA link needs further analysis, given their variable correlation.

CircRNAs are moving towards a better understanding and practical use of their physiological and pathological features in oral medicine, primarily guided by the discovery that they function as miRNA sponges. This review aims to create the framework for a deeper understanding of the diagnosis, prognosis and treatment of oral diseases and offer insight into the functions of circRNAs involved in orthodontics and in other aspects of oral health.

## Data Availability

Not applicable.
